# Urea-to-Albumin Ratio and In-Hospital Mortality in Severe Pneumonia Patients

**DOI:** 10.1155/2021/5105870

**Published:** 2021-10-22

**Authors:** Yu Tian, Yihao Li, Zixin Jiang, Jieru Chen

**Affiliations:** ^1^Department of Intensive Care Unit, The Second Affiliated Hospital, Guangzhou Medical University, Guangzhou, China; ^2^Department of Anesthesiology, The Second Affiliated Hospital, Guangzhou Medical University, Guangzhou, China

## Abstract

**Objective:**

The urea-to-albumin ratio (UAR), as a new marker of the systemic inflammatory response, is associated with the mortality in pneumonia patients. However, the association between the UAR and in-hospital mortality in severe pneumonia (SP) has received little attention.

**Methods:**

In this single-center retrospective cohort study, 212 SP patients in intensive care unit (ICU) from June 1, 2016, to June 1st, 2020, with baseline UAR were enrolled. The primary outcome was in-hospital mortality. The association of UAR with in-hospital mortality was assessed using a multivariable-adjusted Cox model.

**Results:**

Of 212 patients, the median age was 73.0 (61.0, 82.8) years, 70.8% of patients were male, and the APACHE II score was 20.0 (16.0, 26.0). During the hospital period, 101 (47.6%) patients died. In-hospital mortality rates for the lower and higher UAR were 16 (27.6%) and 85 (55.2%), respectively (*P* < 0.001). Kaplan–Meier analysis revealed that survival rates were significantly different between the two groups (log rank = 13.71, *P* < 0.001). After adjusted for confounding factors, the higher UAR group was significantly associated with a hazard ratio (HR) for in-hospital mortality of 2.234 (95% confidence interval: 1.146–4.356, *P*=0.018). Besides, this pattern persisted in subgroup analyses considering sex (HR = 9.380; 95% CI: 2.248–39.138; *P*=0.002).

**Conclusions:**

Higher UAR levels at the commencement of admission to ICU may be independently associated with increased in-hospital mortality in SP patients.

## 1. Introduction

Severe pneumonia (SP) in ICU patients represents a major concern for physicians because of the high mortality and morbidity rate attributable to these episodes [1–3]. Both community-acquired pneumonia or nosocomial pneumonia can progress to SP, which is associated with a mortality rate of more than 50% [4]. During the past decades, many strategies have been implemented with the aim to predict pneumonia severity and prognosis.

Urea-to-albumin ratio (UAR) is a new, inexpensive, and reproducible maker of systemic inflammatory responses that are suitable for routine use and can be easily calculated and determined under simple laboratory conditions. Previous studies reported that a high blood urea nitrogen-to-albumin ratio (B/A) is relevant to critical illness [5]. Risk factors for poor outcomes in patients with CAP include higher blood urea nitrogen and lower albumin [6–10]. B/A levels have also been reported to be associated with a high risk of 30-day mortality in ventilator-associated pneumonia (VAP) patients [11]. Moreover, Mahmood Y et al. applied elevated urea and decreased albumin to COVID-19 pneumonia patients to predict the admission to ICU [5]. However, there is no study on SP patients.

Therefore, in the present study, we aim to assess the association between UAR and in-hospital mortality in SP patients.

## 2. Materials and Methods

### 2.1. Study Design and Population

In this retrospective and cohort study, we enrolled the SP patients between June 1st, 2016, and June 1st, 2020, in the ICU of the Second Affiliated Hospital of Guangzhou Medical University after obtaining institutional approval. Written informed consent was approved by the retrospective nature. Patients who were admitted to ICU were screened and, if eligible, were included. We screened the patients 18 years of age or older who were admitted to the ICU for SP. Patients were excluded for the reasons: (1) ICU duration <24 h, (2) end-stage renal failure (on dialysis), and (3) chronic liver disease. The end-stage renal failure, also known as end-stage renal disease (ESRD), is defined as requiring renal replacement therapy according to kidney disease improving global outcomes (KDIGO) guidelines. Chronic liver disease was defined as one of the following conditions: chronic HBV infection, chronic HCV infection, alcoholic liver disease, nonalcoholic fatty liver disease, and liver cirrhosis due to any cause [12].

### 2.2. Definitions

To confirm the reported clinical SP, the events were defined in a standardized approach with the use of criteria from the guidelines of SP in China (2016 version). Pneumonia was diagnosed when met one of the first four criteria and criteria 5: (1) new cough or the ordinary respiratory disease worsened, with sputum and/or chest pain or not, (2) fever, (3) pulmonary moist rale and/or consolidation, (4) peripheral blood leucocyte count >10 × 10^9^/L or < 4 × 10^9^/L with a nuclear shift to the left or not, and (5) new chest radiographic infiltrate with pleural effusion or not and less possibility of alternative diagnoses. Pneumonia patients were diagnosed with SP when met one of the major criteria or three of the minor criteria. The major criteria include the following: (1) invasive mechanical ventilation and (2) septic shock needing vasopressors. The minor ones are as follows: (1) respiratory rate ≥ 30 breaths/min, (2) multilobar infiltrates, (3) PaO_2_/FiO_2_ ratio ≤ 250, (4) uremia (BUN level > 20 mg/dL), (5) confusion/disorientation, (6) leukopenia (WBC count < 4 × 10^9^/L), (7) thrombocytopenia (platelet count < 100 × 10^9^/L), (8) hypothermia (core temperature < 36°C), and (9) hypotension requiring massive fluid resuscitation.

### 2.3. Data Collection and Outcome

Baseline demographic data included age, sex, hospital-acquired pneumonia (HAP), hypertension, chronic heart disease, diabetes mellitus, stroke, chronic obstructive pulmonary disease, and chronic kidney disease. Clinical and biochemical data at the initial admission to ICU included mean arterial blood pressure (MAP), heart rate (HR), respiratory rate (RR), acute physiology and chronic health evaluation II (APACHE II) score, alanine aminotransferase (ALT), aspartate aminotransferase (AST), creatinine, urea, albumin, white blood cell (WBC), neutrophil count, lymphocyte count, monocyte count, platelet count, red blood cell (RBC), hemoglobin, and hematocrit (HCT). Samples of peripheral blood were stored by tubes with ethylenediamine tetraacetic acid. Primary outcome was in-hospital mortality.

### 2.4. Statistical Analyses

Eligible patients were divided into two groups (UAR ≤ 0.2555 group and UAR > 0.2555 group) by the optimal value of UAR for predicting in-hospital mortality. The receiver operating characteristic (ROC) curve was used to examine the predictive power of UAR and to acquire UAR cutoff values to maximize sensitivity and specificity. Data were expressed as percentages, mean ± standard deviation, median (25–75th percentile). Independent two-group comparisons were performed using the Student *t*-test or Mann–Whitney *U* test for continuous data and chi-square test or Fisher's exact test for categorical data. The cumulative incidence of survival was estimated by using the Kaplan–Meier curve, and differences were compared with the log rank test. The associations between UAR and the primary outcome were evaluated by Cox proportional hazards regression. A univariate Cox regression analysis was conducted to test the correlation between patients' characteristics and in-hospital mortality. Then, the multivariate Cox regression was conducted to evaluate variables significantly associated with the primary outcome, which adjusted for covariates (*P* < 0.05 in univariate Cox analysis or clinical concern (sex)). Moreover, a formal test of interaction between UAR and sex was performed. In Cox models, time at risk was from the study entry until death during hospitalization, transfer, or discharge. The data missing under 5% were replaced by the mean or median. Statistical analyses were performed with SPSS, version 22.0. A value of *P* < 0.05 was considered statistically significant. Figures were conducted using GraphPad Prism Software 9.0.

## 3. Results

### 3.1. Baseline Characters

**Figure 1:**
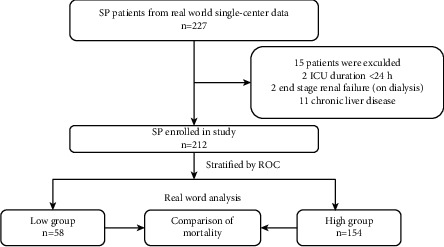
Study algorithm, including patient enrollment and outcomes. Note: low group: UAR ≤ 0.2555; high group: UAR > 0.2555; SP: severe pneumonia; ROC: receiver operating characteristics curve.

From 1st June 2016 to 30th June 2020, a total of 227 SP patients were screened in the ICU, of whom 2 patients whose ICU stay times were less than 24 hours, 2 patients who were at the end stage of renal failure (on dialysis), and 11 patients who have chronic liver disease. The remaining 212 patients with UAR values measured at baseline were eventually enrolled in the study (Figure 1). Baseline characteristics of these patients, categorized based on the optimal value of UAR for predicting in-hospital mortality, are given in Table 1. Of these 212 cases, the median age was 73.0 (61.0, 82.8), 70.8% of the patients were male, and 16.0% were hospital-acquired pneumonia. The median APACHE II score was 20.0 (16.0, 26.0) within the 24 hours after ICU admission. The baseline characteristics were compared between the higher and lower UAR groups. Patients in the higher UAR group were older (*P*=0.001), were more likely to be male (*P*=0.017), and had a higher APACHE II score (*P* < 0.001) than those in the lower UAR group. The radiological findings showed that 82.5% of the patients had bilateral pneumonia and 31.1% had pleural effusion. No significant difference in the radiological findings was observed (*P*=0.389; *P*=0.494, respectively).

### 3.2. Risk Factors for In-Hospital Mortality

All significant factors identified as predictors of in-hospital mortality (*P* < 0.05 in the Cox univariate regression analysis and clinical concern (sex)) are given in Table 2. Then, multivariate Cox analyses identified two prognostic factors for in-hospital mortality, including vasopressor use and CRRT (*P*=0.004; *P*=0.041; respectively).

### 3.3. Performance of Baseline UAR as a Predictor of In-Hospital Mortality by ROC Curve Analysis

**Figure 2:**
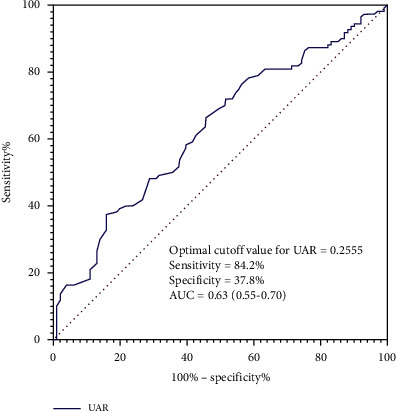
ROC curve for predicting mortality in patients with SP. UAR had a modest power for predicting in-hospital mortality as suggested by AUC of 0.63 (95% CI: 0.55–0.70; *P*=0.001), with a sensitivity of 84.2% and a specificity of 37.8% at a cutoff of 0.2555.

The results of ROC analysis for UAR in predicting in-hospital mortality are shown in Figure 2. It suggested that UAR had a modest power for predicting in-hospital mortality (AUC = 0.63; 95% CI: 0.55–0.70; *P*=0.001). The optimal cutoff value of the UAR for predicting in-hospital mortality was 0.2555 (sensitivity 84.2%; specificity 37.8%).

### 3.4. UAR Associated with In-Hospital Mortality

**Figure 3:**
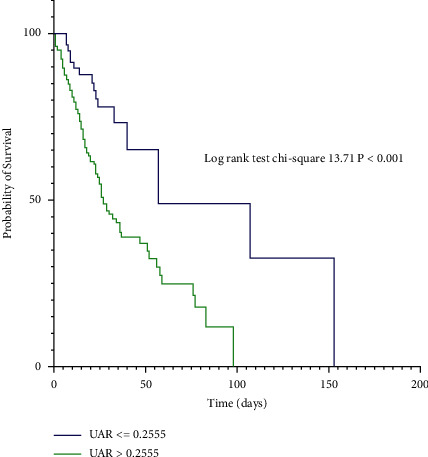
Kaplan–Meier survival curve according to the UAR level. Compared to the lower group (UAR ≤ 0.2555), patients in the higher group (UAR > 0.2555) showed elevated in-hospital mortality.

During follow-up, 101 (47.6%) patients died during the hospitalization. The in-hospital mortality rates for the lower and higher UAR were 27.6% and 55.2%, respectively (Table 1). There were significant differences in the in-hospital mortality rates between the two groups (*P* < 0.001). The Kaplan–Meier estimates of the in-hospital mortality for patients with different UAR values are shown in Figure 3. The Kaplan–Meier estimates revealed that the higher UAR group had higher in-hospital mortality rate than the lower group (log rank = 13.71, *P* < 0.001).

To elucidate the specific relationship between UAR and in-hospital mortality, we used different models (Table 3, UAR ≤ 0.2555 as the reference group). Crude Cox model analysis showed that an increased UAR was a significant predictor of in-hospital mortality (HR = 2.788; 95% CI: 1.577–4.929; *P* < 0.001; model 1). In model 3, after adjusted for age, sex, invasive mechanical ventilation, CRRT, vasopressor use, creatinine, alanine aminotransferase, and aspartate aminotransferase, an increased UAR was still an independent predictor of in-hospital mortality (HR = 2.234; 95% CI: 1.146–4.356; *P*=0.018).

### 3.5. Relationship between Mortality and UAR in the Sex Subgroup

Results of interaction analysis between UAR and sex are given in Table 4. There was a significant interaction between UAR and sex on in-hospitality mortality (*β* = 4.290; *P*=0.004). Thus, a sex-stratified analysis was conducted. In the female subgroup, Cox analysis showed a higher in-hospital mortality in the UAR > 0.2555 group, after adjusted for age, sex, invasive mechanical ventilation, CRRT, vasopressor use, creatinine, alanine aminotransferase, and aspartate aminotransferase (HR = 9.380; 95% CI: 2.248–39.138; *P*=0.002). However, this pattern was not observed in the male subgroup (*P*=0.112).

## 4. Discussion

Our results suggested that the first UAR values after admitted to ICU were independently associated with an increased risk of in-hospital mortality in SP patients. Interestingly, this study found that similar trends were observed in the female subgroup, but not in males. Additionally, patients with higher baseline UAR values may be elderly and more likely to be male and have a higher APACHE II score.

SP, as a severe lung infection and inflammation, is still a common reason for ICU admission with a mortality rate of more than 50%. The UAR, novel inflammatory markers, reflect two pathways, which are probably less affected by confounding conditions and may be more predictive in evaluating the prognosis of pneumonia than urea or albumin separately [13]. Besides, as a simple and objective severity indicator for pneumonia, UAR is easy to implement in routine clinical practice when compared with the pneumonia severity index (PSI) and the CURB-65 score for pneumonia severity [14]. The performance of predictive function about UAR, PSI, and the CURB-65 score was not significantly different [15].

Study has shown that higher urea and lower albumin indicated worse clinical outcome in CAP patients [6–10]. Ugajin et al. revealed that the blood urea nitrogen/serum albumin (B/A) ratio performed well for predicting mortality and severity of CAP [16]. Feng et al.'s study showed that the B/A ratio was associated with poorer survival outcomes in 30-day ventilation-acquired pneumonia (VAP). An earlier study calculated the optimal cutoff point of B/A values for 30-day mortality using ROC curves in CAP patients. The point was at 0.165 [17]. Ryu et al. found that, in aspiration pneumonia patients, the AUC for B/A ratio was at 0.70 for predicting mortality within 28 days [18]. However, very few studies in the literature have evaluated UAR as a predictor of worse outcomes in SP patients. Thus, according to the previous research studies, we speculate that the UAR may be an important indicator of mortality in SP patients. The results of our study were consistent with this speculation.

Our study included both CAP and HAP patients in the ICU. In our ROC curve analysis, we determined a cutoff value of 0.2555 for in-hospital mortality. Multivariable Cox regression identified that the higher UAR was significantly associated with an HR of 2.234 for in-hospital mortality even after adjusted for age, sex, invasive mechanical ventilation, CRRT, vasopressor use, creatinine, alanine aminotransferase, and aspartate aminotransferase.

However, the underlying mechanism has remained unclear. Urea is a marker associated with systemic disease. High values of urea lead to high susceptibility to infection. Some previous studies suggested that urea affects the prognosis of critical patients regardless of the creatine level [19, 20]. In the predictive models, urea is a significant risk factor for pneumonia. Moreover, urea is an indirect marker of a metabolic systemic pathway [21]. In pneumonia patients, elevated values of serum urea are an indicator of protein catabolism. Additionally, water deficiency appears to be common in pneumonia patients. In the process of dehydration, the concentration of urea increased. Meanwhile, the effect of increased urea reabsorption in the kidney causes high urea concentration [18]. Finally, the urea level is regarded as a predictive marker reflecting the cumulative effects of hemodynamic damage, which is essential in critical illness.

Serum albumin plays a significant role in maintaining physiological homeostasis, including keeping a colloid osmotic pressure [22]. On the other hand, hypoalbuminemia can result in the pulmonary edema due to decreased colloid osmotic pressure which can result in mortality [23]. Xue et al. suggested that hypoalbuminemia in the early stage had a high incidence of infection and mortality [24]. At the same time, pneumonia is an inflammation with high catabolism condition. Systemic inflammatory response can decrease serum albumin levels [22]. Clearly, hypoalbuminemia is often observed in malnutrition patients, resulting in worse outcomes. It is interesting to note that earlier studies mostly focused on CAP showed that nonsurvivors have significantly lower urea and higher albumin than those of survivors. The study reported before demonstrated that UAR is an independent marker of the severity of CAP and mortality [22]. Our findings are consistent with previous conclusions.

Further subgroup analyses were conducted by Kaplan–Meier analysis. Another notable finding was that higher UAR were associated with a higher risk of cumulative in-hospital mortality in female patients. To the authors' knowledge, this research may be the first time to reveal the association between UAR and in-hospital mortality in female patients. A previous study reported that males had a higher fractional synthesis rate of albumin than females regardless of age and protein intake. Males had higher albumin concentration than females [25]. Therefore, the increase of UAR in critical illness was not so obvious in males. Weaving et al. demonstrated that albumin values in females decreased more quickly [26]. This is owing to the different values of parameters between males and females. Our results are consistent with the previous studies. Further studies are needed to examine why UAR is associated with mortality in female patients.

Our research has some limitations. First, as a single-center retrospective study, it may contain bias and be insufficient to draw the same conclusion in other populations. Second, the samples were small, so the predictive value of the UAR needs to be further validated in other observational studies. Third, in the present study, we investigated only baseline variables rather than changes over time in these variables in SP patients who were admitted to the ICU. Besides, we did not detect different types of pneumonia and its relationship with the mortality. The next generation sequencing (NGS) test will be added in our future study for better verification of conclusion.

## 5. Conclusions

Our study demonstrated that UAR is an independent risk factor for in-hospital mortality in SP patients, especially in females. These findings suggested that the baseline UAR, a simple and widely available biomarker, could be useful for risk stratification of in-hospital mortality in SP patients. We found this conclusion in our preprint [27].

## Figures and Tables

**Table 1:** Comparison of baseline characteristics.

Variables	Total (*n* = 212)	UAR ≤ 0.2555 (*n* = 58)	UAR > 0.2555 (*n* = 154)	*P* value
Demographic data				
Age (years)	73.0 (61.0, 82.8)	66.0 (53.8, 78.3)	78.5 (63.0, 84.0)	0.001
Sex (male)	150, 70.8%	34, 58.6%	116, 75.3%	0.017
HAP	34, 16.0%	8, 13.8%	26, 16.9%	0.585
Underlying diseases				
Hypertension	119, 56.1%	21, 36.2%	98, 63.6%	<0.001
Diabetes mellitus	53, 25.0%	10, 17.2%	43, 27.9%	0.109
CHD	25, 11.8%	4, 6.9%	21, 13.6%	0.175
Stroke	44, 20.8%	8, 13.8%	36, 23.4%	0.125
COPD	27, 12.7%	4, 6.9%	23, 14.9%	0.118
CKD	27, 12.7%	1, 1.7%	26, 16.9%	0.003
Radiological findings				
Bilateral pneumonia	175, 82.5%	50, 86.2%	125, 81.2%	0.389
Pleural effusion	66, 31.1%	16, 27.6%	50, 32.5%	0.494
Treatment				
Invasive mechanical use	109, 51.4%	34, 58.6%	75, 48.7%	0.294
Vasopressor use	73, 34.4%	14, 24.1%	59, 38.3%	0.053
CRRT	73, 34.4%	7, 12.1%	66, 42.9%	<0.001
Clinical data				
MAP (mmHg)	83.0 (69.5, 103.0)	85.0 (74.6, 108.5)	83.0 (66.9, 101.3)	0.153
Heart rate (rate/min)	109.9 (83.8, 136.0)	104.5 (84.4, 124.6)	111.9 (84.1, 139.7)	0.035
Respiratory rate (rate/min)	27.0 (22.0, 34.8)	25.5 (20.0, 33.3)	27.0 (22.0, 35.0)	0.161
APACHE II score	20.0 (16.0, 26.0)	16.5 (12.0, 20.0)	22.0 (17.0, 27.3)	<0.001
Laboratory results				
ALT (U/L)	34.5 (21.0, 65.8)	33.0 (21.0, 51.3)	34.8 (21.0, 76.8)	0.265
AST (U/L)	44.5 (27.0, 82.0)	42.5 (24.5, 60.8)	44.5 (28.0, 97.0)	0.054
Creatinine (umol/L)	114.9 (72.8, 191.4)	65.2 (51.1, 85.6)	140.4 (98.0, 239.0)	<0.001
Urea (mmol/L)	11.8 (6.9, 19.1)	5.5 (4.2, 6.5)	15.4 (11.1, 21.6)	<0.001
Albumin (g/L)	29.0 (24.1, 33.9)	30.8 (26.5, 35.1)	28.3 (23.3, 33.3)	0.001
WBC (10^9^/L)	11.7 (8.0, 16.0)	12.9 (9.9, 16.8)	11.2 (6.8, 15.5)	0.035
Neutrophil count (10^9^/L)	10.0 (6.3, 13.9)	11.0 (8.4, 14.6)	9.7 (5.4, 13.8)	0.072
Lymphocyte count (10^9^/L)	0.6 (0.3, 1.0)	0.8 (0.4, 1.3)	0.6 (0.3, 0.9)	0.006
Monocyte count (10^9^/L)	0.5 (0.2, 0.8)	0.6 (0.3, 1.1)	0.5 (0.2,0.8)	0.008
Platelet count (10^9^/L)	205.0 (117.3, 280.5)	234.5 (176.8, 322.0)	186.5 (98.3, 256.0)	<0.001
RBC (10^12^/L)	3.6 (2.6, 4.6)	4.0 (3.1, 4.9)	3.4 (2.4, 4.4)	<0.001
Hemoglobin (g/L)	102.9 (73.8, 132.0)	112.9 (89.2, 136.6)	99.1 (69.0, 129.2)	0.001
HCT (%)	31.3 (22.7, 39.9)	33.9 (26.6, 41.2)	30.3 (21.4, 39.2)	0.004
Clinical outcomes				
ICU LOS (days)	12.0 (6.3, 21.0)	14.0 (9.0, 24.3)	11.0 (5.0, 19.3)	0.020
Hospital LOS (days)	21.0 (11.3, 32.0)	25.0 (15.5, 36.3)	18.0 (10.0, 32.0)	0.040
In-hospital death	101, 47.6%	16, 27.6%	85, 55,2%	<0.001

Data are mean ± standard or medians (25th–75th percentile) or number and percentage. UAR, urea-to-albumin ratio; HAP, hospital-acquired pneumonia; CHD, coronary heart disease; COPD, chronic obstructive pulmonary disease; CKD, chronic kidney disease; CRRT, continuous renal replacement therapy; MAP, mean arterial pressure; APACHE, acute physiology and chronic health evaluation; ALT, alanine aminotransferase; AST, aspartate aminotransferase; WBC, white blood cell; RBC, red blood cell; HCT, hematocrit; LOS, length of stay.

**Table 2:** Independent predictors of in-hospital mortality by univariate and multivariate Cox regression analyses.

Factors	HR (95% CI)	*P*
Univariate Cox analysis		
Age	1.013 (1.001–1.026)	0.029
Mechanical ventilation	0.796 (0.641–0.990)	0.040
Vasopressor use	2.407 (1.619–3.578)	<0.001
CRRT	2.402 (1.607–3.592)	<0.001
ALT	1.001 (1.000–1.001)	0.006
Albumin	0.947 (0.908–0.988)	0.011
Urea	1.020 (1.003–1.037)	0.024
AST	1.001 (1.000–1.001)	<0.001
Creatinine	1.002 (1.000–1.003)	0.016
Multivariate Cox analysis		
Vasopressor use	1.888 (1.226–2.907)	0.004
CRRT	1.679 (1.020–2.762)	0.041

Covariates included in multivariate analysis: age, sex, mechanical ventilation, vasopressor use, CRRT, ALT, albumin, urea, AST, creatinine. CRRT, continuous renal replacement treatment; ALT, alanine aminotransferase; AST, aspartate aminotransferase; HR, hazard ratio; CI, confidence interval.

**Table 3:** Relationship between UAR level and in-hospital mortality.

In-hospital mortality	UAR >0.2555 group	
	HR (95%CI)	*P*
Unadjusted	2.788 (1.577–4.929)	<0.001
Model 1	2.724 (1.499–4.949)	0.001
Model 2	2.080 (1.100–3.934)	0.024
Model 3	2.234 (1.146–4.356)	0.018

Reference group is UAR ≤ 0.2555 group. Model 1: age and sex. Model 2: model 1 plus treatment (mechanical ventilation, CRRT, and vasopressor use). Model 3: model 2 plus and laboratory test (creatinine, ALT, and AST). CRRT, continuous renal replacement treatment; ALT, alanine aminotransferase; AST, aspartate aminotransferase; HR, hazard ratio; CI, confidence interval.

**Table 4:** Relationship between in-hospital mortality and UAR level by sex.

In-hospital mortality	Male		Female		Sex^*∗*^U/A interaction	
	HR (95% CI)	*P*	HR (95% CI)	*P*	*β*	*P*
UAR	0.520 (0.232–1.165)	0.112	9.380 (2.248–39.138)	0.002	4.290	0.004

Adjusted for age, mechanical ventilation, CRRT, vasopressor use, ALT, AST, creatinine. CRRT, continuous renal replacement treatment; ALT, alanine aminotransferase; AST, aspartate aminotransferase; HR, hazard ratio; CI, confidence interval.

## Data Availability

The data used to support the findings of this study are available from the corresponding author upon request.
